# *N*-*tert-*butylmethanimine *N-*oxide is an efficient spin-trapping probe for EPR analysis of glutathione thiyl radical

**DOI:** 10.1038/srep38773

**Published:** 2016-12-12

**Authors:** Melanie J. Scott, Timothy R. Billiar, Detcho A. Stoyanovsky

**Affiliations:** 1 Department of Surgery, University of Pittsburgh, Pittsburgh, Pennsylvania, USA; 2 Department of Environmental and Occupational Health, University of Pittsburgh, Pittsburgh, Pennsylvania, USA

## Abstract

The electron spin resonance (EPR) spin-trapping technique allows detection of radical species with nanosecond half-lives. This technique is based on the high rates of addition of radicals to nitrones or nitroso compounds (spin traps; STs). The paramagnetic nitroxides (spin-adducts) formed as a result of reactions between STs and radical species are relatively stable compounds whose EPR spectra represent “structural fingerprints” of the parent radical species. Herein we report a novel protocol for the synthesis of *N*-*tert*-butylmethanimine *N-*oxide (EBN), which is the simplest nitrone containing an α-H and a tertiary α′-C atom. We present EPR spin-trapping proof that: (i) EBN is an efficient probe for the analysis of glutathione thiyl radical (GS^•^); (ii) β-cyclodextrins increase the kinetic stability of the spin-adduct EBN/^•^SG; and (iii) in aqueous solutions, EBN does not react with superoxide anion radical (O_2_^−•^) to form EBN/^•^OOH to any significant extent. The data presented complement previous studies within the context of synthetic accessibility to EBN and efficient spin-trapping analysis of GS^•^.

The electron spin resonance (EPR) spin-trapping technique is an analytical method that allows detection of radical species with nanosecond half-lives. This technique is based on the high rates of addition of radicals (X^•^) to nitrones (Fig. 1, **1**) or nitroso compounds (spin traps; STs)^1^,^2^,^3^. The paramagnetic nitroxides (spin-adducts; **2**) formed as a result of reactions between STs and radical species are relatively stable compounds (t_1/2_(spin-adducts) = seconds − hours) whose EPR spectra represent “structural fingerprints” of the parent radical species. To date, over 100 nitrones have been assessed as STs^4^,^5^ and the NIH spin-trapping database contains more than 10,000 entries from experiments performed with approximately 20 STs (http://tools.niehs.nih.gov/stdb/).

Analysis of EPR spectra and the stability of analogous series of spin-adducts indicates that cyclic STs with an α−H and a tertiary α′−C atom (Fig. 1, denoted in red and blue color, respectively) tend to form nitroxides with more resolved EPR spectra than their acyclic analogues^4^ and that hindrance of the nitroxide group stabilizes^6^,^7^,^8^ spin-adducts while polarization of the N-Cα bond destabilizes them^9^,^10^. The sensitivity of the spin-trapping technique is negatively affected by the dismutation of α-H spin-adducts to nitrones and hydroxylamines^8^,^11^, whereas analyses in biological matrices are further complicated by the propensity of nitroxides to undergo one-electron reduction or oxidation either to “EPR-silent” hydroxylamines or to oxoammonium salts^12^,^13^,^14^. The short half-lives of most spin-adducts necessitate the performance of analyses under steady-state conditions in which radical species are generated at considerable rates. Hence, there is a continuous effort to enhance the sensitivity of the EPR spin-trapping technique via identification of nitrones that form spin-adducts with increased stability.

In this paper, we report the spin-trapping analysis of selected biologically-relevant radical species by *N*-*ter*t-butyl(methylideneamine) *N*-oxide (EBN; Fig. 1). We provide experimental proof that EBN reacts with glutathione thiyl radical (GS^•^) to form EBN/^•^SG, which exhibits a distinct EPR Spectrum. We further show that EBN/^•^SG is a relatively stable nitroxide as compared to spin-adducts of GS^•^ with a number of widely used STs, and that β- and β-methyl-cyclodextrin (β-CD and β-Me-CD) extend the analytical window for assessment of GS^•^ by increasing the kinetic stability of EBN/^•^SG.

## Results and Discussion

### Synthesis of EBN

EBN, the simplest nitrone containing an α-H and a tertiary α′-C atom, has been extensively used as a reagent for cycloaddition reactions^15^,^16^,^17^. In early spin-trapping studies with nitroso compounds, Chalfont *et al*. noted that EBN can be used as an alternative ST for detection of carbon-centered radicals^18^,^19^. However, EPR spin-trapping data obtained with this nitrone have not been reported thus far.

Coupling either of 2-methyl-2-nitroso-propane with diazomethane^20^ (CAUTION, highly toxic compound) or of aqueous formaldehyde with *N-tert-*butylhydroxylamine (BHA)^21^ affords EBN in good to excellent yields. Following the latter protocol, we attained vacuum distillation of EBN, but failed to obtain a nitrone fraction that was free of trace amounts of nitroxides, which ultimately interfere with EPR spin-trapping experiments. Purification of the nitrone by activated charcoal or by column chromatography also proved difficult as the end reaction products exhibited comparable polarity. Hence, we optimized the synthetic protocol via assessment of the effects of solvents and the source of formaldehyde on the yield of EBN. EPR-grade EBN was obtained in quantitative yield via treatment of BHA hydrochloride with an excess of paraformaldehyde in CH_2_Cl_2_, as described in Methods.

### Spin-trapping of GS^•^ by EBN

The metabolism of redox-sensitive xenobiotics often proceeds with generation of free radicals, which, in turn, react with thiols to form thiyl radicals. As glutathione is the most abundant cellular thiol, its oxidation by free radicals to GS^•^ is a preponderant reaction, and the formation of GS^•^ is viewed as a toxicological event as this radical species abstracts H atoms from cellular molecules, reacts with sulfhydryls to form disulfides, and adds to double bonds^22^,^23^.

**Figure 1:**
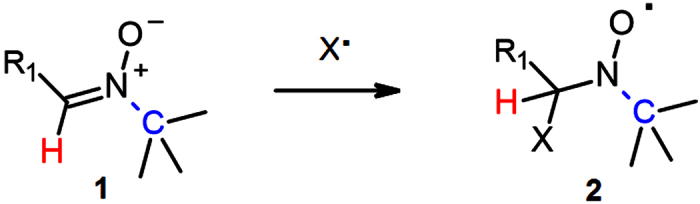
Nitrones react with radicals to form nitroxides.

**Figure 2:**
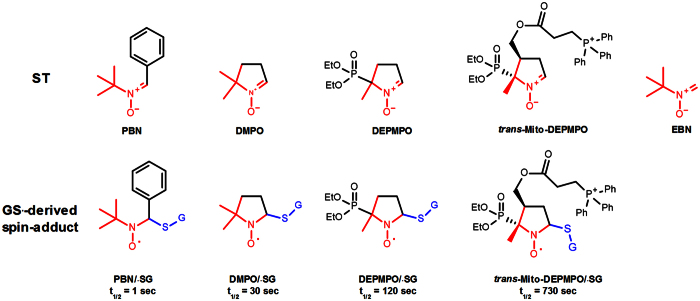
Structures of selected STs and their spin-adducts with GS^•^.

The detection of GS^•^ in biological matrices is difficult because its half-life is in the nano-to micro-second scale^24^. Research in the 1980s demonstrated that GS^•^ reacts with 5,5-dimethyl-1-pyrroline *N*-oxide (DMPO) to form DMPO/^•^SG (Fig. 2), which exhibits a specific four-line EPR spectrum^25^,^26^,^27^,^28^. While this protocol proved instrumental in the elucidation of fundamental redox reactions of GSH, its application is limited by the low stability of DMPO/^•^SG (t_1/2_ ≃ 50 s)^28^,^29^,^30^,^31^. Recent analyses of the kinetics of formation and decay of a number of GS^•^-derived spin-adducts have identified 5-(diethoxyphosphoryl)-5-methyl-1-pyrroline-N-oxide (DEPMPO) and *trans*-Mito-DEPMPO as STs that form kinetically more stable spin-adducts with GS· than DMPO (Fig. 1)^32^. To extend the structure-activity relationship study of the spin-trapping analysis of GS^•^, we have carried out experiments with EBN, which is a common structural motif of a number of widely-used STs (Fig. 2; common bonds in nitrones are denoted in red).

**Figure 3:**
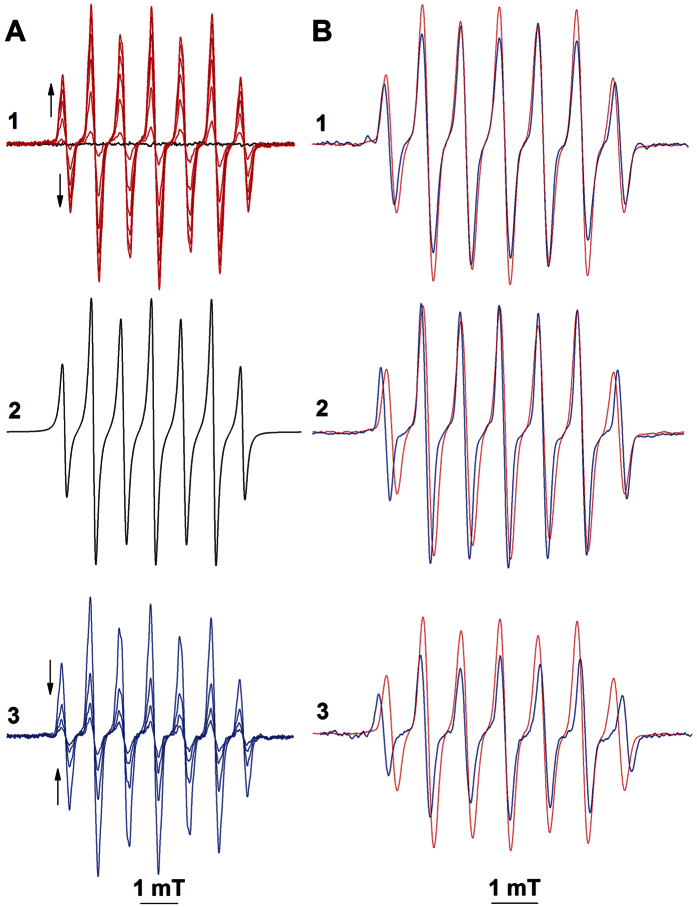
Spin-trapping of photolytically generated thiyl radicals by EBN. Reactions were carried out at 25 °C in 0.1 M phosphate buffer (pH 7.4) containing EBN (50 mM) and *S-*nitrosothiol (5 mM). **A1**- EPR spectra of GSNO and EBN prior to (light off; black trace) and after illumination (light on; red traces); **2**- computer simulation of the EPR spectrum of EBN/^•^SG; **3**- EPR-monitored decay of EBN/^•^SG (light off). Consecutive spectra were recorded with time intervals of 30 s (**1**) and 150** **s (**3**). Arrows indicate the directions of the spectral changes. **B**- EPR spectra were recorded after illumination of *S*-nitrosothiols and EBN for 5 min; **1**- *S-*nitroso cysteine and EBN; **2** *S-*nitroso *N*-acetyl-D-penicillamine and EBN; *S-*nitroso 2-methyl-2-propanethiol and EBN. EPR spectra were recorded at an Amplification of 100.

The data presented in Fig. 3A show the spin-trapping of GS^•^ with EBN. We generated GS^•^ via photolytic homolysis of the S-N bond of *S*-nitrosoglutathione (GSNO)^30^. At ambient luminance (<400 lux), the reaction system consisting of GSNO and EBN did not exhibit any EPR activity (Fig. 3A1, black trace). Irradiation of the solution with visible light (515 nm cutoff filter; 1 × 10^5^ Lux) led to the appearance of a seven-line EPR spectrum with hyperfine splitting constants (in mT) of a_H_ = 0.729 and a_N_ = 1.607, which was assigned to EBN/^•^SG (Fig. 3A1, red tracings). In Fig. 3A2 is presented a computer simulation of the EPR spectrum of EBN/^•^SG. When GS^•^ was spin-trapped by 50 mM EBN in the presence of 100 mM and 200 mM DMPO, the magnitude of the EPR spectrum of EBN/^•^SG decreased by 33% and by 66%, respectively, indicating that K^SG^_EBN_ ≃ 1.5 k^SG^_DMPO_ (data not shown; comparison of the rate constants were made as reported in ref. 33; K^SG^_DMPO_ = 1 × 10^8^ M^−1^s^1^)^10^.

The reaction solution containing EBN and GSNO was illuminated until EBN/^•^SG reached a steady-state concentration, after which interruption of the illumination led to a continuous decrease of the EPR spectrum with an apparent t_1/2_^(EBN/SG)^ of 120 s (Fig. 3A3, blue lines). No changes in either the formation or the decay of EBN/^•^SG were observed in the pH interval of 5 to 8, nor in the presence of up to 1 M NaCl or LiClO_4_, indicating that these processes do not dependent on ionic interactions (data not shown).

In Fig. 3B we present the EPR spectra of the spin-adducts of EBN with the thiyl radicals of L-cysteine, *N*-acetyl-D-penicillamine and 2-methyl-2-propanethiol, which were generated photolytically from the corresponding *S*-nitrosothiols (EBN/^•^SCys, EBN/^•^SNAP, and EBN/^•^SBu; blue lines, traces 1 (a_H_ = 0.811 and a_N_ = 1.651), 2 (a_H_ = 0.884 and a_N_ = 1.722) and 3 (a_H_ = 0.897 and a_N_ = 1.762), respectively). The spectra of EBN/^•^SG (red traces, B1-3), EBN/^•^SNAP and EBN/^•^SBu were readily distinguishable (Fig. 3B2 and B3). The spectra of EBN/^•^SG and EBN/^•^SCys were similar but exhibited different intensity patterns (Fig. 3B1); for EBN/^•^SG, spectral maxima 3 and 5 were smaller than 4 and 6, whereas this ratio was reversed for EBN/^•^SCys. These data indicate that EBN can be used as a spin-trapping probe for identification of thiyl radicals with different substituents in Cα relative to the sulfur atom.

### Stabilization of EBN/^•^SG by cyclodextrins (CDs)

**Figure 4:**
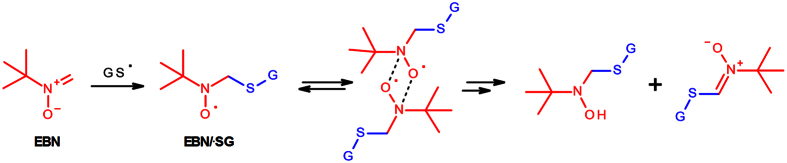
Nitroxides containing α-H atoms dismutate to nitrones and hydroxylamines.

The dismutation of α-H nitroxides to nitrones and hydroxylamines is a major reaction pathway leading to the decay of spin-adducts. The reaction proceeds via formation of a nitroxide-dimer wherein a single electron transfer from nitrogen to oxygen yields the ion pair “hydroxylamine anion/oxoammonium cation”; tautomerization of the oxoammonium cation, with concomitant release of a proton, leads to the formation of a new nitrone (Fig. 4; reviewed in ref. 34).

One strategy to increase the kinetic stability of spin-adducts containing α-H atoms is to impede their dimerization via inclusion into CDs. Cyclodextrins are cyclic polymers consisting of 6, 7 or 8 glucopyranoside units (α-CD, β-CD or γ-CD) which have the shape of toroids (7.9 Å), with the larger and smaller openings exposed to the solvent. They have hydrophilic interfaces and hydrophobic cavities with diameters of 4.7, 6, and 7.5 Å (α−, β− and γ−CD, respectively). CDs have a propensity for forming non-covalent inclusion complexes with a variety of hydrophobic organic molecules^35^,^36^, including nitroxides^37^,^38^,^39^,^40^. The spin-trapping of superoxide anion radical (O_2_^−•^) with a series of STs in the presence of β-Me-CD has been shown to proceed with inclusion of the corresponding spin-adducts in the cavity of the cyclodextrin, thus increasing their half-lives^41^,^42^,^43^,^44^,^45^,^46^.

**Figure 5:**
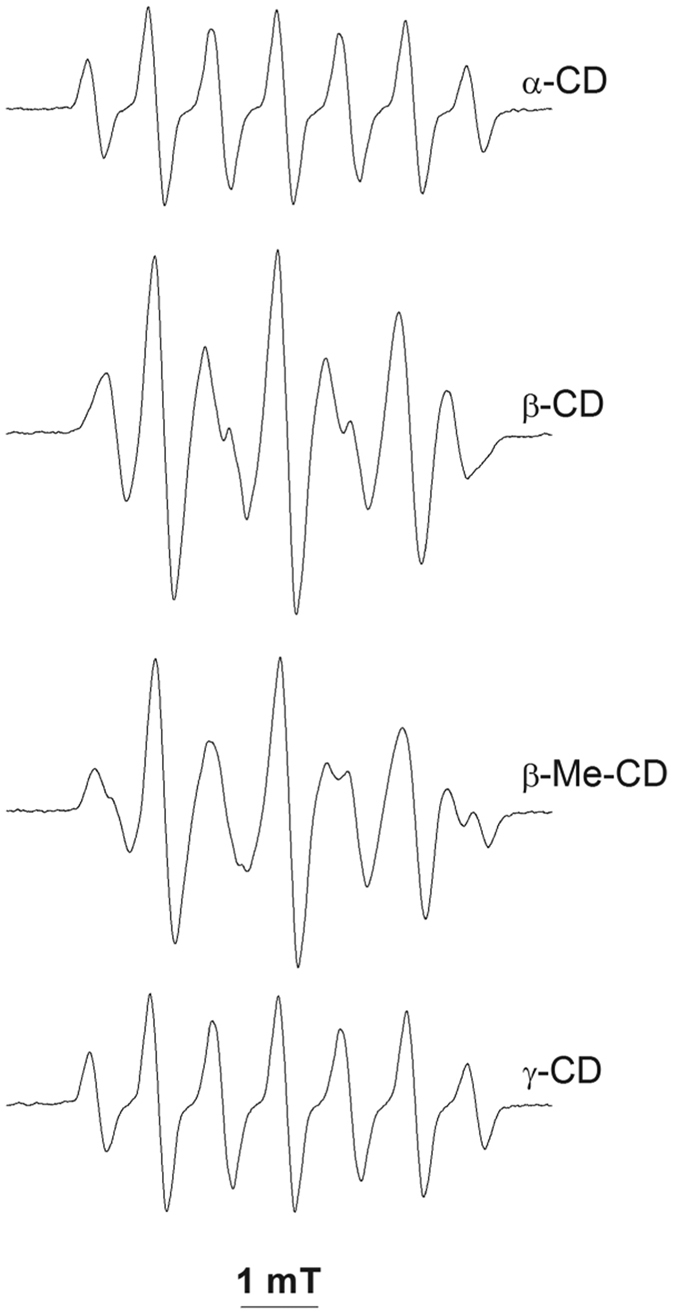
Effects of CDs on the EPR spectrum of EBN/^•^SG. EPR spectra (Amplification, 500) were recorded after photolytic homolysis of GSNO (5 mM) for 3 min in the presence of EBN (15 mM) and CDs (15 mM). All other incubation conditions are the same as indicated in the legend to Fig. 2.

The EPR spectra presented in Fig. 5 were obtained with generation of EBN/^•^SG in the presence of α-, β-, β-Me-, and γ-CD. In all experiments, EBN and CDs were used at 15 mM concentration (saturated solution of β-CD, 17 mM). As compared to the spectrum of EBN/^•^SG, new EPR-active species were formed in the presence of β- and β-Me-, but not in the presence of α- and γ-CD. We observed the same spectral changes upon addition of CDs to pre-formed EBN/^•^SG (data not shown). Although marked spectral changes were detected in the hyperfine splitting constants of EBN/^•^SG upon its inclusion in CDs, resolved signals from the free and bound nitroxide were not obtained, and hence we did not assess the constants of the corresponding inclusion complexes. In the presence of β-CD, the EPR signal of EBN/^•^SG increased linearly with increases in the concentration of GSNO (0.005–0.2 mM). Since α- and γ-CD differ from β- and β-Me-CD only in the sizes of their cavities, spectral changes due to surface adsorption of the nitroxide on the outside of the cavities and/or spin-trapping of secondary radicals generated by reactions between GS^•^ and/or EBN/^•^SG with CDs can be excluded. Furthermore, since β-CD has a hydrophobic channel with a diameter of 6 Å, inclusion of the whole EBN/^•^SG radical can be ruled out as well. The decrease in a_N_ in the presence of β-CDs indicates that the nitroxide function was compartmentalized in a more hydrophobic milieu, suggesting that the *tert*-butyl side of the spin-adduct was included in the CDs, whereas the hydrophilic, glutathionyl part of the molecule remained exposed to the bulk water.

**Figure 6:**
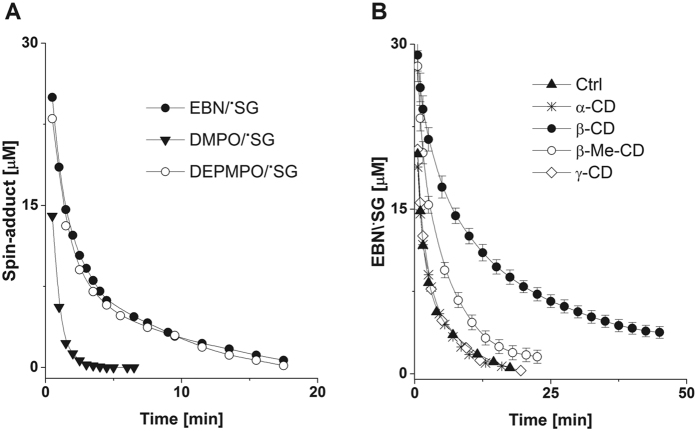
Effects of CDs on the decay of EBN/^•^SG. Photolytic homolysis of GSNO to GS^•^ was carried out for 3 minutes in the presence of DMPO, DEPMPO and EBN. Thereafter, the light was switched off and decreases in the EPR spectra of spin-adducts were recorded over time in the absence (**A**) and the presence (**B**) of CDs (15 mM). Nitrones were used at concentrations of 50 mM (**A**) and 15 mM (**B**). The spin concentration of nitroxides was determined by double integration of the EPR signals using 4-hydroxyl-1-TEMPO as a standard. All other reaction conditions are the same as indicated in the legend to Fig. 2. The data in panels B are presented as mean values of three independent experiments ± the standard error.

In Fig. 6A, we show comparative kinetics of the decays of EBN/^•^SG, DMPO/^•^SG, and DEPMPO/^•^SG. The apparent t_1/2_ values of 50 and 120 seconds for DMPO/^•^SG and DEPMPO/^•^SG were in good agreement with previous studies^29^,^30^,^32^, and the decay of EBN/^•^SG and DEPMPO/^•^SG exhibited similar kinetic profiles. Next, we assessed the kinetics of decay of EBN/^•^SG in the presence of CDs (Fig. 6B); in the presence of β-CD, the apparent t_1/2_ of EBN/^•^SG increased from 120 s to 450 s, and the spectrum of the spin-adduct was readily detectable 90 min after interruption of the photolytic generation of GS^•^. Although EBN/^•^SG formed inclusion complexes with both β- and β-Me-CD, the stability of the spin-adduct in the presence of β-Me-CD was lower (t_1/2_ = 190 s). This suggests that either the rate of release of EBN/^•^SG from its complex with β-Me-CD was higher than from β-CD, presumably due to decreased hydrogen bonding between methylated OH groups and the nitroxide, or to the methyl groups obstructing its inclusion in the cyclodextrin.

### Spin-trapping of O_2_^−•^ and hydroxyl radical (HO^•^) by EBN

A considerable research effort has been directed toward the identification of STs that can be used for analysis of O_2_^−•^ and HO^•^, which are radical species of considerable importance as reaction intermediates in various biological, radiolytic, and photochemical processes. In 1974, Harbour *et al*. reported that, in aqueous solutions, O_2_^−•^ can be spin-trapped by DMPO^47^. Although this method has been extensively used, it has considerable analytical limitations; the reaction of DMPO with O_2_^−•^ is rather slow (k^Superoxide^_DMPO_ = 10 M^−1^s^−1^), DMPO/^•^OOH is an unstable spin adduct (t_1/2_ = 50 s), and the analysis is affected by trace amounts of transition metal ions and pH variations^33^,^48^. In 1995, Frejaville *et al*. found that DEPMPO, an α-phosphorus-containing analogue of DMPO, reacts with O_2_^−•^ to form DEPMPO/^•^OOH, which is 15 times more stable than DMPO/^•^OOH^33^. Rosen *et al*. observed that increases in the bulkiness of alkyl substituents in third position of the pyrroline ring of DMPO leads to complete inhibition of the spin-trapping of O_2_^−•^ 49, as indicated by the formation of the corresponding nitroxides, while Allouch *et al*. found that introduction of electron-withdrawing groups in Cα position of acyclic STs inhibits the spin-trapping of O_2_^−•^ 50. In a theoretical analysis of cyclic STs, Villamena *et al*. reported that the electronic density on the nitronyl C atom noticeably changes with introduction of substituents in C_α−δ_ and thus affects the reactions of nitrones with O-centered radicals. However, these studies have not been extended to a structure-activity relationship that allows prediction of the spin-trapping affinity of nitrones for specific radical species.

**Figure 7:**
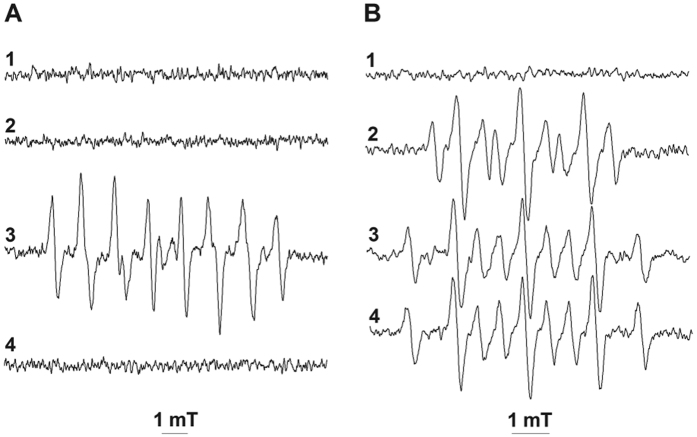
Spin-trapping of O_2_^−•^, HO^•^ and CH_3_^•^ by EBN. Reactions were carried out in 0.1 M phosphate buffer (pH 7.4) containing EDTA (0.05 mM). **A1**- EBN (50 mM), catalse (300 U/ml) and KO_2_ (0.2 mM); **A2**- EBN, catalase, HX (0.5 mM) and XO (50 mU/mL); **A3**- DEPMPO (20 mM), catalase, HX and XO; **A4**- DEPMPO, catalase, HX, XO and SOD (30 U/mL). **B1**- H_2_O_2_ (0.05 mM) and EBN (20 mM); **B2**- plus Fe(NH_4_)_2_SO_4_ (0.01 mM); **B3**- DMSO (0.2 M), EBN and H_2_O_2_ plus Fe(NH_4_)_2_SO_4_; **B4-** authentic EBN/^•^CH_3_,. EPR spectra were recorded at an Amplification of 1000.

Our attempts to obtain the spin-adduct of EBN with O_2_^−•^ in aqueous solutions proved unsuccessful. In phosphate buffer (0.1 M; pH 7.4) containing catalase (300 U/mL) and EBN (50–200 mM), we did not observe the formation of EPR-active species upon addition of up to 0.4 mM KO_2_ (Fig. 7A1; stock solution of KO_2_ was prepared in DMSO containing an equimolar amount of 18-Crown 6). Similarly, we did not observe formation of EBN/^•^OOH when O_2_^−•^ was enzymatically generated by the system hypoxanthine (HX; 0.5 mM)/xanthine oxidase (XO; 50 mU/mL; Fig. 7A2). Substitution of EBN with DEPMPO in the HX/XO system resulted in the appearance of the typical EPR spectrum of DEPMPO/^•^OOH (Fig. 7A3), thus indicating that O_2_^−•^ was generated in the reaction solution. Superoxide dismutase (SOD; 30 U/mL) fully inhibited the formation of DEPMPO/^•^OOH (Fig. 7A4). In contrast to O_2_^−•^, the generation of HO^•^ in a Fenton-like system containing EBN led to the appearance of a well resolved nine-line EPR spectrum, which we assigned to EBN/^•^OH (Fig. 7B2; in mT, a_H_ = 0.613 and a_N_ = 1.125; H_2_O_2_ + Fe^2+^ → HO^•^ + Fe^3+^ + HO^−^; EBN + HO^•^ → EBN/^•^OH). No EPR activity was observed if any one of the reagents was omitted from the reaction system (Fig. 7B1). Introduction of DMSO in the reaction system led to the formation of EBN/^•^CH_3_, which exhibited a distinct EPR spectrum (Fig. 7B3; CH_3_S(O)CH_3_ + HO^•^ → CH_3_^•^ + CH_3_S(O)OH; EBN + CH_3_^•^ → EBN/^•^CH_3_). The identity of the latter nitroxide was confirmed by EPR analysis of authentic EBN/^•^CH_3_, which was synthesized by methylation of EBN with CH_3_MgI (Fig. 7B4; in mT, a_H_ = 1.199 and a_N_ = 1.854).

**Figure 8:**
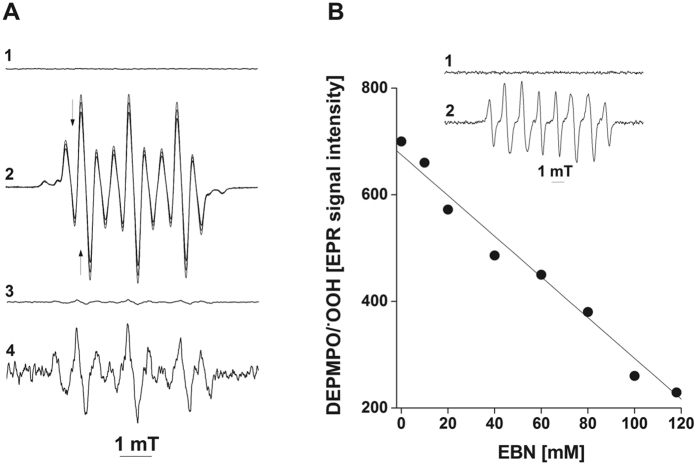
Spin-trapping of O_2_^−•^ by EBN in DMSO and by DEPMPO and EBN in aqueous solutions. **A1**- EBN (20 mM) in DMSO containing 0.05 mM H_2_O; **A2**- EBN, KO_2_ (0.4 mM) and 18-Crown 6 (0.4 mM; Δ_scan_ = 2 min; arrows indicate the direction of spectral changes); **A3**- EBN/^•^OOH (0.02 mL) plus phosphate buffer (pH 7.4; 0.08 mL; Amplification, 100); **A4**- the same spectrum as in A3 but recorded at an Amplification of 4000. **B**- Effects of EBN on the spin-trapping of O_2_^−•^ by DEPMPO. Reactions were carried out at 25 °C in 0.1 M phosphate buffer (pH 7.4) containing catalase (300 units/mL). Inset, **1**- DEPMPO (10 mM); **2**- DEPMPO and 18-Crown 6/KO_2_ (0.1 mM). Spectra were recorded at an Amplification of 1000.

We further carried out experiments to assess the reaction of EBN with O_2_^−•^ in an aprotic solvent. Addition of a solution of 18-Crown 6/KO_2_ (final concentration, 0.4 mM) in anhydrous DMSO to DMSO containing EBN (20 mM) and H_2_O (0.05 mM; Fig. 8A1) led to the appearance of a nine-line EPR spectrum, reflecting the formation of EBN/^•^OOH (Fig. 8A2; in mT, a_H_ = 0.483 and a_N_ = 0.975). The magnitude of the EPR signal decreased by 15% for 6 min, indicating that EBN/^•^OOH was unstable under these reaction conditions. An identical EPR spectrum was observed when a solution of EBN in DMSO was treated with H_2_O_2_ (20 mM) and Et_3_N (100 mM), thus supporting the assignment of the EPR spectrum to EBN/^•^OOH; in the latter reaction system, a nucleophylic addition of HOO^−^ to the nitronyl C atom yielded EBN/OOH hydroxylamine, which autooxidized to EBN/^•^OOH nitroxide (data not shown). Dilution of a DMSO solution of EBN/^•^OOH with phosphate buffer (pH 7.4) resulted in the disappearance of the EPR spectrum of EBN/^•^OOH (Fig. 8A3) in less than 30 seconds, which is the approximate time required for sample preparation and data acquisition. However, we did observe the presence of trace amounts of a relatively stable nitroxide, less than 5% of the expected concentration of EBN/^•^OOH, with hyperfine coupling constants suggesting the formation of EBN/^•^OH (Fig. 8A3 and 4- Amplification 100 and 4000, respectively).

In spin-trapping experiments, nitroxides are analyzed by EPR under steady-state conditions, where their rates of formation and decomposition define the analytical sensitivity of the corresponding protocol. While the data presented in Fig. 8A indicate that EBN/^•^OOH is an unstable compound in aqueous solutions, we were interested to assess the rate of the reaction of EBN and O_2_^−•^ in phosphate buffer (pH 7.4). To this end, we carried out a competitive kinetics study of the reaction of DEPMPO with O_2_^−•^ in the presence of EBN, where the source of O_2_^−•^ was 18-Crown 6/KO_2_. Addition of O_2_^−•^ (final concentration, 0.1 mM) to a solution of DEPMPO (10 mM) in 0.1 M phosphate buffer (Fig. 8B; Inset, trace 1) resulted in the formation of DEPMPO/^•^OOH (Fig. 8B; Inset, trace 2). The magnitude of the EPR signal of DEPMPO/^•^OOH decreased linearly with increasing concentrations of EBN, with 50% inhibition of the formation of DEPMPO/^•^OOH at ~100 mM EBN. These data indicate that the rate constant of the reaction of EBN with O_2_^−•^ is one order of magnitude lower than that of DEPMPO (k^Superoxide^_DEPMPO_ = 15 M^−1^s^−1^)^33^. Hence, in contrast to DMPO and its analogues, EBN is a suitable ST for analysis of O_2_^−•^ formed in organic but not in aqueous solutions, where both the rate constant of its reaction with O_2_^−•^ and the stability of EBN/^•^OOH are relatively low.

### Spin-trapping of enzymatically-generated GS^•^ with EBN

**Figure 9:**
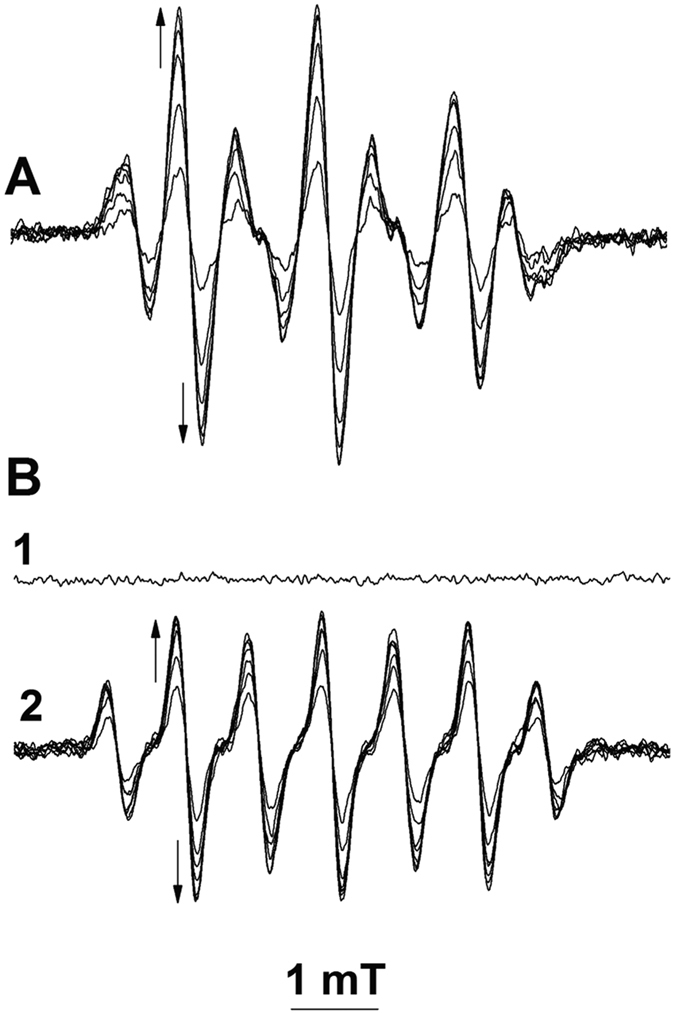
Spin-trapping of enzymatically-generated O_2_^−•^. Reactions were carried out at 25 °C in 0.1 M phosphate buffer (pH 7.4). **A**- EBN (15 mM), β-CD (15 mM), GSH (2 mM), HX (0.5 mM), XO (50 mU/mL), and catalase (300 U/mL). **B1**- Phenol (0.01 mM), MPx (0.2 units/mL), H_2_O_2_ (0.1 mM), and GSH (1 mM); **B2-** Phenol, MPx, H_2_O_2_, GSH and EBN (20 mM). Consecutive spectra were recorded with time interval of 120 s. Arrows indicate the directions of the spectral changes. EPR spectra were recorded at an Amplification of 1000.

The low reactivity of EBN with O_2_^−•^ in aqueous solutions suggests that this nitrone can be used for spin-trapping analysis of secondary, O_2_^−•^-derived radicals in biological systems. This possibility is illustrated by the data presented in Fig. 9A. Generation of O_2_^−•^ by HX/XO in the presence of EBN, GSH, and catalase resulted in the formation of EBN/^•^SG; in neutral aqueous solutions, O_2_^−•^ oxidized GSH to GS^•^ with a rate constant^51^ of 10^3^ M^−1^s^−1^. Addition of SOD to the reaction system fully inhibited the formation of EBN/^•^SG (data not shown). In this system, the spin-trapping selectivity of EBN was reminiscent to the affinity of 2-H-imidazole-1-oxide for thiyl radicals but not for O_2_^−•^ 52.

**Figure 10:**
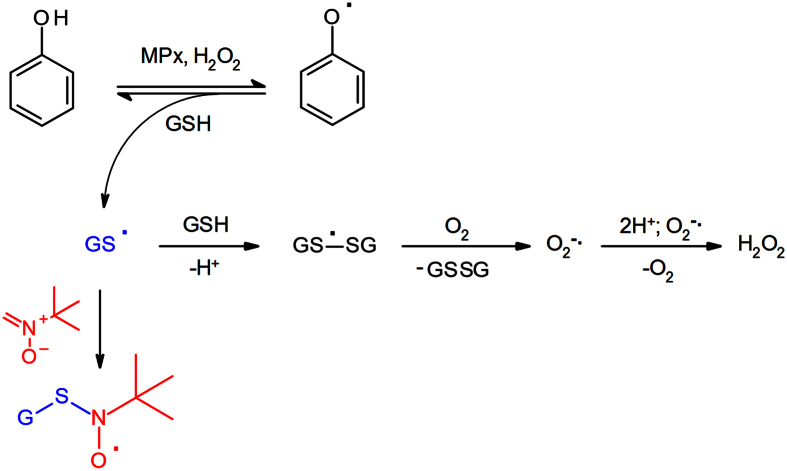
Phenol phenoxyl radical oxidizes GSH to GS^•^.

To further validate EBN as a spin-trapping probe for GS^•^, we assessed the formation of GS^•^ in a reaction system consisting of myeloperoxidase from human leucocytes (MPx; 0.2 units/mL), phenol (0.01 mM), GSH (1 mM), and H_2_O_2_ (0.1 mM). In this system, phenol undergoes oxidation to phenoxyl radical, which is reduced back to phenol by GSH with concomitant generation of GS^•^; in turn, GSH reacts with the latter to form a disulfide anion radical which transfers an electron to O_2_, yielding GSSG and O_2_^−•^ 53. In cells, this reaction sequence can occur without apparent consumption of phenol and GSH, whose concentration is maintained via reduction of GSSG by glutathione reductase (Fig. 10).

In the absence of EBN, the complete reaction system did not exhibit any EPR activity (Fig. 9B1), indicating that the concentration of radical species was below the detection limit of the EPR spectrometer. Addition of EBN led to the appearance of the EPR spectrum of EBN/^•^SG, whose intensity increased until a steady-state concentration of EBN/^•^SG has been reached. Elimination of H_2_O_2_ by addition of catalase to the reaction system led to disappearance of the EPR signal of EBN/^•^SG with a kinetic profile that was identical to that presented in Fig. 6A (data not shown).

## Conclusions

The data presented herein complement previous studies within the context of synthetic accessibility to EBN and efficient spin-trapping analysis of GS^•^. From all nitrones tested thus far, *trans*-Mito-DEPMPO, DEPMPO and EBN form the most stable spin adducts with GS^•^ (t_1/2_^*trans*^-Mito-DEPMPO/SG = 730 sec; t_1/2_^DEPMPO/SG^ = 120 sec; and t_1/2_^EBN/SG^ = 120 sec). As compared to EBN, however, the synthesis of EPR grade DMPO analogues requires higher experimental effort. The relatively high rate of addition of GS^•^ to EBN, the kinetic stability of EBN/^•^SG, and the well-resolved EPR spectrum of EBN/^•^SG define this nitrone as an efficient molecular probe for GS^•^.

## Materials and Methods

### Reagents

All reagents used were purchased from Sigma-Aldrich Co. (St. Louis, MO). *N-tert*-butylhydroxylamine was synthesized as described in ref. 54. The solutions used in EPR-STs experiments were prepared in deionized and Chelex 100-treated water and in potassium phosphate buffer (pH 7.4). *S*-nitroso thiols were prepared by treatment of thiols with ethyl nitrite as reported in ref. 55. Methylation of EBN with CH_3_MgI was carried out as reported in ref. 56.

#### Synthesis of N-tert-butylmethanimine oxide (EBN)

Under an atmosphere of helium, a suspension of BHA hydrochloride (0.65 g; 5.2 mmol), paraformaldehyde (0.46 g; 15.3 mmol), anhydrous Na_2_SO_4_ (3.5 g; 24.6 mmol) and NaHCO_3_ (2.5 g; 30 mmol) in 30 mL CH_2_Cl_2_ was stirred at 25 °C for 5 hours. Progression of the reaction was tracked via HPLC analysis of EBN. Chromatographic separations were carried out on a C18 matrix (column, Beckman; 4.6 × 250 mm; particle size, 5 μ) with methanol (40%) as the mobile phase (flow rate, 1 mL per min). Formation of EBN (retention time, 5.6 min; λ_max_ = 230 nm; peak purity, 100%; software, EZchrom 4.3) was monitored with a SPD M10Avp Shimadzu diode array detector. Upon completion of the reaction, the suspension was filtered and the filtrate rotor-evaporated (30 °C; 250 torr), affording EBN as a colorless liquid (yield, 100%). MS m/z, calculated for C_5_H_12_NO (M)^+^ 102.09, found 101.88. Analogous yields of EBN were obtained with BHA actetate, which is a commercially available salt (Sigma-Aldrich, Co; St. Louis, MO). Following the protocol reported in reference 21, EPR-grade EBN was obtained via purification of the crude product by column chromatography (50 mg product on 150 g Silica Gel 60; mobile phase, 10% methanol in diethyl ether (v/v); yield, 45%).

#### Photolysis of S-nitrosothiols

Visible (white) light was provided by a Sylvania lamp type DWY (625 W) equipped with a spherical reflector. The light source was positioned 30 cm from the front of the EPR cavity, where Teflon tubing containing a solution of GSNO, EBN and DTPA (0.1 mM) in 0.1 M phosphate buffer (pH 7.4) was placed. The aperture of the cavity was equipped with a 515 nm cut-off filter (colored glass filter OG515; Melles Griot BV; Aalsbergen, Nederland). Light intensity was measured with a Datalogging Light Meter (Model 850008; SPER Scientific, LTD; Scottsdale, AZ).

#### EPR Spectroscopy

EPR spectra were recorded at room temperature using a JEOL-RE1X spectrometer (Kyoto, Japan). Spectrometer settings were:  field center 335.094 mT, microwave power 10 mW, sweep time 30–120 s, time constant 0.1 s, and modulation width 0.2 mT. EPR spectra simulations were performed with a JEOL computer program.

## Additional Information

**How to cite this article:** Scott, M. J. *et al. N*-*tert*-butylmethanimine *N*-oxide is an efficient spin-trapping probe for EPR analysis of glutathione thiyl radical. *Sci. Rep.* **6**, 38773; doi: 10.1038/srep38773 (2016).

**Publisher's note:** Springer Nature remains neutral with regard to jurisdictional claims in published maps and institutional affiliations.
